# IGTCM: An integrative genome database of traditional Chinese medicine plants

**DOI:** 10.1002/tpg2.20317

**Published:** 2023-03-09

**Authors:** Yuan‐Nong Ye, Ding‐Fa Liang, Jia‐Hao Yi, Shuai Jin, Zhu Zeng

**Affiliations:** ^1^ Cells and Antibody Engineering Research Center of Guizhou Province Key Laboratory of Biology and Medical Engineering School of Biology and Engineering Guizhou Medical University Guiyang China; ^2^ Bioinformatics and Biomedical Big Data Mining Laboratory Department of Medical Informatics School of Big Health Guizhou Medical University Guiyang China

## Abstract

Fully understanding traditional Chinese medicines (TCMs) is still challenging because of the extreme complexity of their chemical components and mechanisms of action. The TCM Plant Genome Project aimed to obtain genetic information, determine gene functions, discover regulatory networks of herbal species, and elucidate the molecular mechanisms involved in the disease prevention and treatment, thereby accelerating the modernization of TCMs. A comprehensive database that contains TCM‐related information will provide a vital resource. Here, we present an integrative genome database of TCM plants (IGTCM) that contains 14,711,220 records of 83 annotated TCM‐related herb genomes, including 3,610,350 genes, 3,534,314 proteins and corresponding coding sequences, and 4,032,242 RNAs, as well as 1033 non‐redundant component records for 68 herbs, downloaded and integrated from the GenBank and RefSeq databases. For minimal interconnectivity, each gene, protein, and component was annotated using the eggNOG‐mapper tool and Kyoto Encyclopedia of Genes and Genomes database to acquire pathway information and enzyme classifications. These features can be linked across several species and different components. The IGTCM database also provides visualization and sequence similarity search tools for data analyses. These annotated herb genome sequences in IGTCM database are a necessary resource for systematically exploring genes related to the biosynthesis of compounds that have significant medicinal activities and excellent agronomic traits that can be used to improve TCM‐related varieties through molecular breeding. It also provides valuable data and tools for future research on drug discovery and the protection and rational use of TCM plant resources. The IGTCM database is freely available at http://yeyn.group:96/.

## Core Ideas

- It helps researchers to explore potential mechanisms of action of a gene with ingredients in an herb.
- It would help to identify new candidate compounds for drug developers.
- It provides a theory and platform for the breeding, cultivation, and molecular identification of traditional Chinese medicine (TCM) species.
- It provides a tool for the molecular identity of herb species and the discovery of active ingredients in TCM plants.

**Abbreviations**
BPGD: the Brazilian Pharmacopoeia Genomic Database
GPGD: Global Pharmacopoeia Genome Database
HMOD: Omics Database for Herbal Medicinal Plants
IGTCM: an Integrative Genome Database of TCM plants
NCBI: The National Center for Biotechnology Information
TCM‐BLAST: Traditional Chinese Medicine Basic Local Alignment Search Tool
TCMID: Traditional Chinese Medicine Integrated Database
TCMs: traditional Chinese medicines
TCMSP: Traditional Chinese Medicine System's Pharmacology Database and Analysis Platform

## 1 INTRODUCTION

Plants used in traditional Chinese medicine (TCM) are crucial components in the fields of health and treatment (Gong et al., 2022). Nevertheless, it is difficult to understand TCMs at a deep level because of the extreme complexity in both chemical ingredients and mechanisms of action, and this has limited their expansion and application.

The lack of genomic information on the original herbal species used in TCMs has marginalized their use in modern medicine and seriously restricted their further development (Gan et al., 2021; Sun et al., 2020; Zhao et al., 2021). Herb genomics can help to bridge the gap between traditional herbal medicine and cutting‐edge omics research (Hu et al., 2019; Li & Chen, 2020). Analyses of complete genome sequences can provide genetic information about species origin, evolution, individual development, important agronomic traits, and secondary metabolite biosynthetic pathways. Genomic analyses of medicinal herbal plants can help to clarify the genetic background of the herbs used in TCMs (Rehman et al., 2020) and enable the discovery of valuable resources for investigating novel bioactive compounds (Chen et al., 2019). Such analyses are important for gene discovery, drug discovery, synthetic biology, and molecular breeding (Mochida et al., 2017).

Medicine plants contain complex natural compounds, making them crucial resources for drug discovery and development (Azwanida, 2015). Approximately 30% of therapeutic drugs are estimated to be derived from natural resources, especially plants and microorganisms (Cragg & Newman, 2013; Newman & Cragg, 2012). The discovery of artemisinin (malaria treatment) by Tu Youyou (Callaway & Cyranoski, 2015) and the discovery of salicin (analgesic and antipyretic) from *Salix alba* by Rafaele Piria in 1832 (Dutra et al., 2016) demonstrate the potential roles of medicinal plants. In modern drug discovery and development, the extraction of active ingredients and the use of omics technology to analyze the mechanisms of action of the active ingredients of TCMs have provided new directions for disease treatment (Harvey et al., 2015; Xu et al., 2019). Integrated genome analyses can increase our understanding of the effects of these active ingredients at the molecular level (Lv et al., 2017). For example, connectivity maps have been widely used to identify molecular mechanisms of candidate drugs, repurpose existing drugs, and elucidate the mechanisms of drug action (Jiang et al., 2021; Qu & Rajpal, 2012). Bioinformatics tools and herbal genomics will improve our understanding of the mechanisms of action of herbal species used in TCMs and gradually allow TCMs to be incorporated into modern medicine.

Desired chemical compounds can be synthesized using plant transcriptome and genome sequencing data by bacterial engineering (Smanski et al., 2016; Wang et al., 2018). To date, many ingredients with significant medicinal values have been extracted from plants, including artemisinin (Callaway & Cyranoski, 2015; Klayman, 1985), paclitaxel (Weaver, 2014), and vinblastine (Muniraj et al., 2019). The rapidly accumulating omics data will help to elucidate the biosynthetic pathways related to medicinal compounds and further advance the discovery, development, and synthesis of new drugs (Guo et al., 2018; Xu et al., 2017; Zhou et al., 2021).

**Core ideas**

- It helps researchers to explore potential mechanisms of action of a gene with ingredients in an herb.
- It would help to identify new candidate compounds for drug developers.
- It provides a theory and platform for the breeding, cultivation, and molecular identification of traditional Chinese medicine (TCM) species.
- It provides a tool for the molecular identity of herb species and the discovery of active ingredients in TCM plants.

Many TCM plants are still obtained from endangered wild resources, and many wild herbal species have become endangered because of habitat destruction and excessive and indiscriminate mining (Chik et al., 2015; Mohanty et al., 2013). Although some TCM plants are obtained from wild resources that are not currently endangered, the challenge of resource reduction still exists. Therefore, annotated genome sequences of herbal species are an irreplaceable resource for finding useful genes for the genetic improvement of TCM plants through molecular breeding (Song et al., 2018; Xu et al., 2017; Zhang et al., 2017) and for developing sustainable TCM plant resources (Cordell, 2015). Molecular marker‐assisted breeding based on genomic data will contribute to the sustainable development of wild medicinal plants, greatly reduce breeding time, rapidly enrich germplasm resources, improve herb yields, and effectively protect wild resources (Bevan et al., 2017). Several crop breeding programs have been developed and are in continuous use, such as those for wheat (Alaux et al., 2018) and corn (Portwood et al., 2019), but no database for the development and breeding of TCM plants is currently available.

In the last decade, several TCM plant databases have been established and have undergone continuous stepwise improvements. GenBank contains a large amount of TCM plant genome information, but this information has not been fully organized and effectively used. The number of genome assemblies for medicinal plants continues to increase. Therefore, we collected, organized, and displayed the genomic data of herbs that are used in TCM from GenBank and RefSeq databases. Each TCM ingredient and related gene were annotated using the eggNOG‐mapper tool(Cantalapiedra et al., 2021; Huerta‐Cepas et al., 2019) and Kyoto Encyclopedia of Genes and Genomes (KEGG)(Kanehisa et al., 2017) database to obtain pathway information (e.g., KEGG) and enzyme classifications (EC numbers), which can be linked across several species and different components. These data were integrated into the Integrative Genome Database of Traditional Chinese Medicine (IGTCM) database for further use. The IGTCM database contains genetic information on medicinal herbs and is a valuable resource for accelerating genome research, molecular breeding, and the discovery of useful genes and active TCM ingredients. The IGTCM database also contains the BLAST (Mount, 2007) and JBrowse (Buels et al., 2016) tools to aid in data analyses.

## 2 MATERIALS AND METHODS

### 2.1 Acquisition of TCM‐related genome data and data processing

**FIGURE 1:**
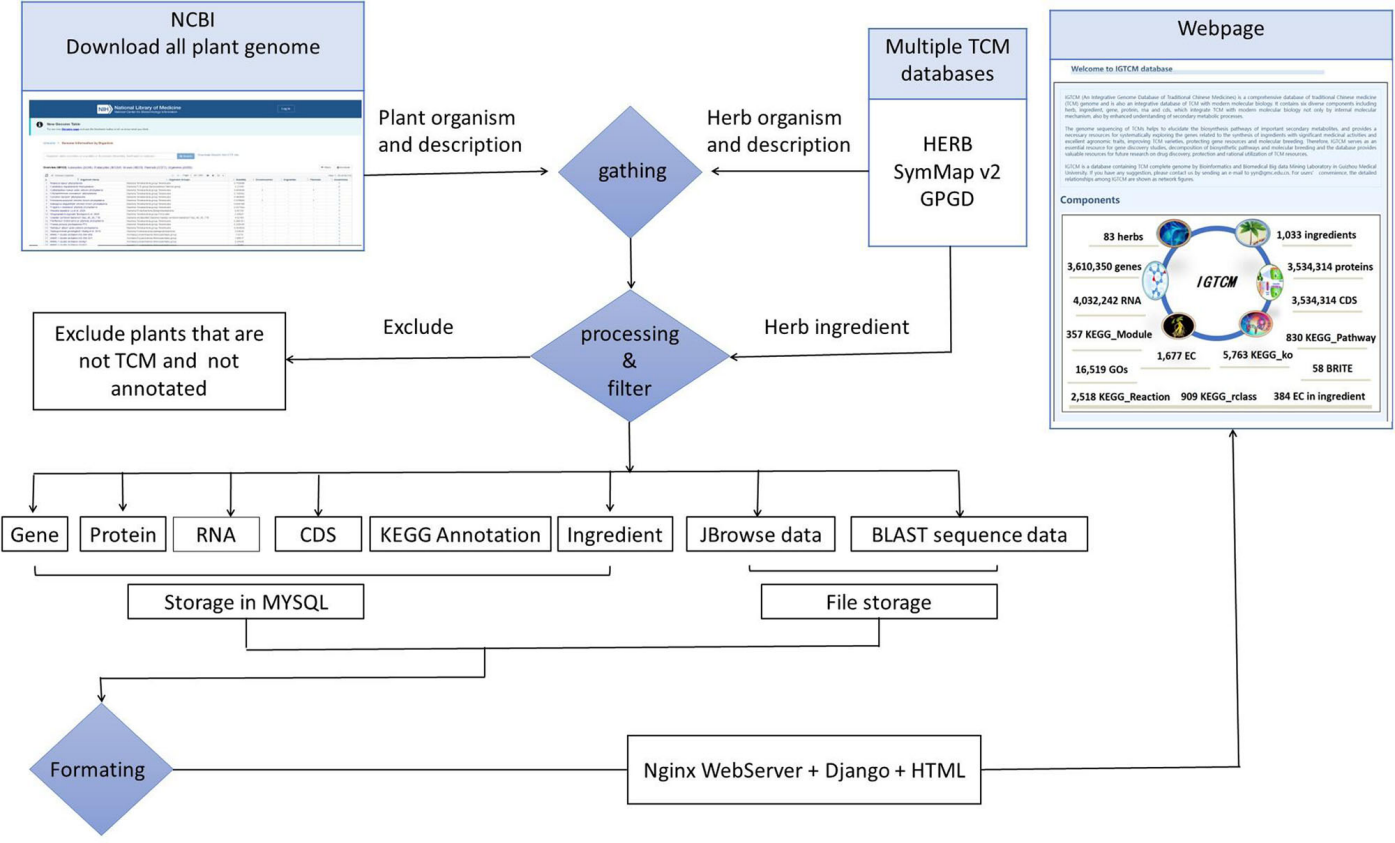
Data collection, processing, and workflow in the IGTCM database. CDS, coding DNA sequence; GPGD, Global Pharmacopoeia Genome Database; IGTCM, Integrative Genome Database of Traditional Chinese Medicine Plants; KEGG, Kyoto Encyclopedia of Genes and Genomes; NCBI, The National Center for Biotechnology Information; TCM, traditional Chinese medicine.

The annotated genome data of 83 herbs used in TCM were downloaded from GenBank and RefSeq databases and were used to build the IGTCM database. The included genome data were uploaded to the NCBI database before October 20, 2021. The latest version of each genome was maintained when multiple versions of the same genome were present. The genome data were categorized as gene, protein, RNA, and coding DNA sequence (CDS). To obtain non‐redundant lists in the same format, we processed the data as follows (Figure 1):

- The gene sequences were extracted mainly from the genome annotation file (GFF) and sequence file (FASTA) (Pearson, 2016). For example, the sequence and annotation information of *LOC113847446* (DNA‐directed RNA polymerase subunit alpha‐like), which is located at 60088–70063 on the negative strand of the *Abrus precatorius* genome (GenBank: NW_020874290.1), was extracted. Then, the protein and CDS sequence, gene length, and GC content were obtained using the Biopython package. Genes that were located on the negative strand were reverse complemented. The correctness of each extraction was verified against the NCBI database.
- The CDS, protein, and RNA sequence were extracted from the rich and comprehensive annotation files provided by the NCBI database and processed to obtain non‐redundant structured data for inclusion in the IGTCM database.
- To further annotate TCM ingredient genomes, we used a high‐throughput tool and pipeline, eggNOG‐mapper, which automatically provides EC, KEGG, BRITE, and GO annotations.

### 2.2 Chinese medicinal herbs and their components

The components of each herb in the IGTCM database were collected from the SymMap v2 (Wu et al., 2019) and HERB (Fang et al., 2021) databases, which provide detailed information, such as the 2D structures, molecule formulae, and the molecular Simplified Molecular Input Line Entry System (SMILES) of the components. This information is also included in PubChem and the National Database for Chemical Composition in TCMs, and it can be considered to be of high quality and have a high application value. Through the molecular formulae of chemical components, all the enzymes involved in the reaction were found in the KEGG database. We integrated the data from these databases and obtained 1033 non‐redundant component records and 1601 enzymes found in 68 herbs for inclusion in the IGTCM database (Figure 1).

### 2.3 Database design and implementation

**FIGURE 2:**
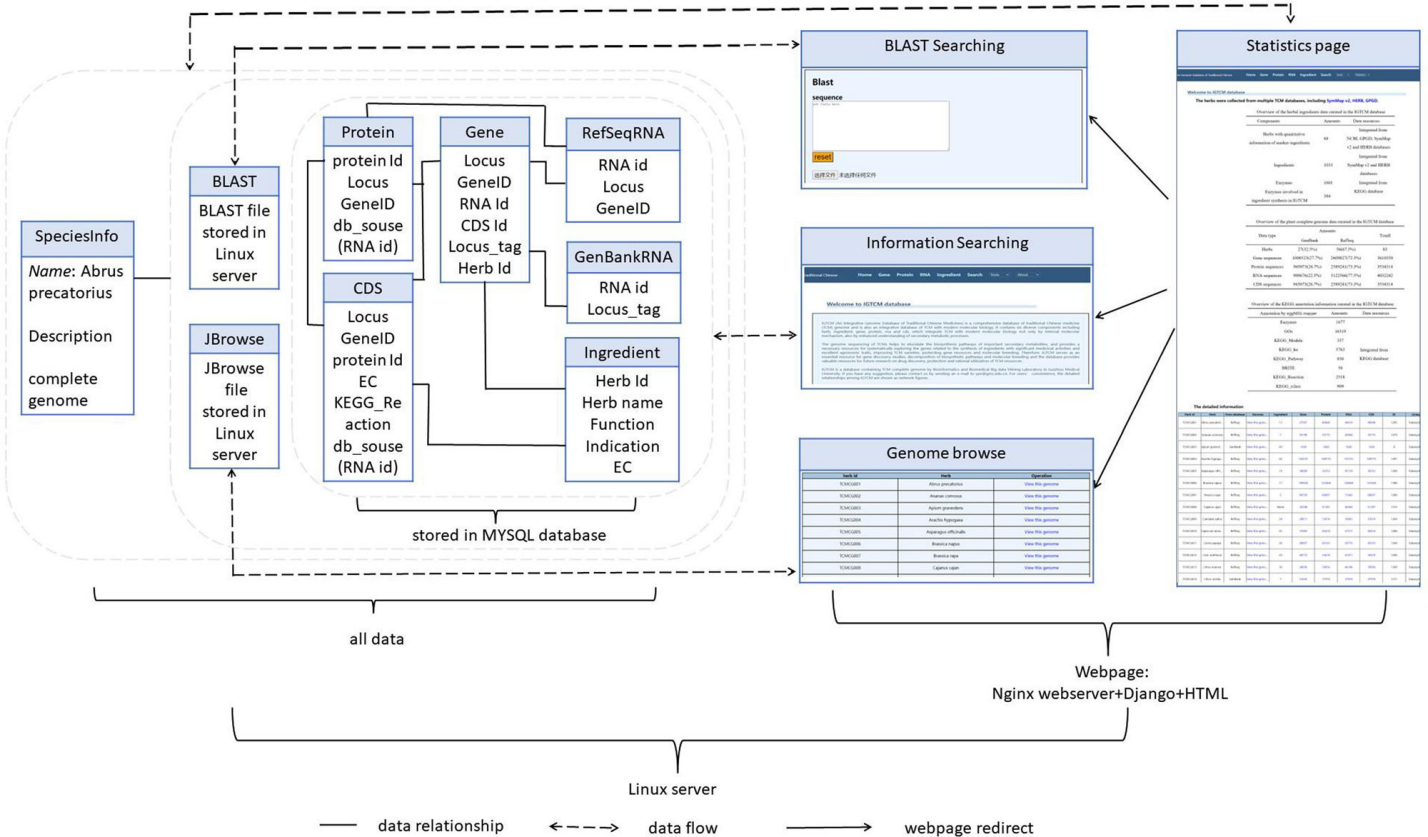
Schematic overview of the Integrative Genome Database of Traditional Chinese Medicine Plants (IGTCM) database construction process. KEGG, Kyoto Encyclopedia of Genes and Genomes.

The IGTCM database is compatible with most major browsers. It was built using Python‐Django, Ajax, Nginx, and HTML (http://www.w3.org) on a Linux server, and the complete genome data are stored in a MySQL relational database (http://www.mysql.com). BLAST and JBrowse are embedded in IGTCM for sequence similarity searches and genome visualization. The IGTCM database is freely accessible at http://yeyn.group:96/. We created six tables, Gene, Protein, GenBankRNA, RefSeqRNA, CDS, and Ingredient, and two file storage servers, BLAST and JBrowse. Their relationships and the construction process are shown in Figure 2. The Gene table contains all the gene sequences and is the core table that connects the other five tables. The 83 complete herbal genomes include 56 that were annotated in RefSeq (O'Leary et al., 2015) and 27 that were annotated in GenBank (Benson et al., 2018). The RNA table also contains RNAs annotated in GenBank and RNAs annotated in RefSeq (Table 1). The Ingredient table contains details for the active components of 68 of the 83 herbs (Table 2). The IGTCM database has a user‐friendly web interface that can be used to browse, search, visualize, and download the data for each herb in the database.

**TABLE 1:** Overview of the plant complete genome data curated in the IGTCM database.

Data type	Count		Total
	GenBank	RefSeq	
Herbs	27 (32.5%)	56 (67.5%)	83
Gene sequences	1000523 (27.7%)	2609827 (72.3%)	3610350
Protein sequences	945073 (26.7%)	2589241 (73.3%)	3534314
RNA sequences	909676 (22.5%)	3122566 (77.5%)	4032242
CDS sequences	945073 (26.7%)	2589241 (73.3%)	3534314

Abbreviations: CDS, coding DNA sequence; IGTCM, Integrative Genome Database of Traditional Chinese Medicine Plants.

**TABLE 2:** Overview of the herbal ingredients data curated in the IGTCM database.

Components	Count	Data resources
Herbs with quantitative information of marker ingredients	68	Integrated from NCBI, GPGD, SymMap v2, and HERB databases
Ingredients	1033	Integrated from SymMap v2 and HERB databases
Enzymes	1601	Integrated from KEGG database
Enzymes involved in ingredient synthesis in IGTCM	384	

Abbreviations: GPGD, Global Pharmacopoeia Genome Database; IGTCM, Integrative Genome Database of Traditional Chinese Medicine Plants; KEGG, Kyoto Encyclopedia of Genes and Genomes; NCBI, National Center for Biotechnology Information.

### 2.4 The association of TCM genomes with each other and with components

In IGTCM implementation, the connectivity among gene, protein, and RNA data was realized using protein and RNA IDs in the Gene table. Conversely, the Protein and RNA tables were also correlated with Gene table through IDs. In addition, in the Protein table, the correlation between protein and RNA data was realized by using the db_source field in the Protein table, and cross‐species correlations were also realized using KEGG annotation information. Finally, in the Ingredient table, the enzymes were used to realize the correlations between the components and protein data. Therefore, the interrelations among different components and cross‐species correlations are realized in IGTCM.

## 3 RESULTS

### 3.1 Genomic and ingredient data stored in the IGTCM database

The IGTCM database contains the annotated complete genomes for 83 herbs used in TCM plants; 27 of the genomes are from GenBank and 56 are from RefSeq. After processing, we obtained 14,711,220 non‐redundant annotation records of 3,610,350 genes, 3,534,314 proteins, 4,032,242 RNAs, and 3,534,314 CDSs (Table 1 and Table S1). By integrating multiple TCM‐related databases and the KEGG database, we obtained 1033 non‐redundant components in 68 herbs and 1601 enzymes involved in the compound synthesis, among which 384 enzymes were involved in IGTCM (Table 2 and Table S1). The IGTCM database also contains the genome sequences from 83 herbs, including gene, protein, RNA, and CDS data that can be used for BLAST searches.

The IGTCM database contains important novel features that are not part of previous TCM plant databases. The power of the IGTCM database lies in achieving minimal inter‐operability for gene, protein, RNA, and CDS data, as well as across several species, using pathway information (KEGG_Pathway and KEGG_Reaction) and enzyme classifications (EC). It also allows linkage to associated genes with (some) compounds in the ingredient list through EC classification. Therefore, users can search for a gene of interest and download related proteins, CDSs, RNAs, and molecular information or KEGG annotation information. BLAST and JBrowse, which are implemented in the IGTCM database, can then be used for further analyses (Figure 2). All of the complete genome and component data in IGTCM are organized and stored in a MYSQL relational database or file storage server on a Linux server, and they can be freely queried and downloaded.

### 3.2 KEGG annotations of TCM genomes

There is also a substantial information loss in the NCBI database. For example, gene *LOC113848124* in the TCM *A. precatorius* has only sequences and a cryptic description, and the encoded protein is not functionally annotated. To compensate for this deficiency and further annotate the TCM genome, we used a high‐throughput tool and pipeline, eggNOG‐mapper, which automatically provides EC, KEGG, BRITE, and GO annotations for each gene. The KEGG COMPOUND database was used to acquire enzymes involved in the compounds found in the ingredient tables. We obtained non‐redundant information, including 1677 enzymes, 16,519 gene ontology classifications, 58 BRITEs, 357 KEGG modules, 5763 orthologs, 830 pathways, 2518 reactions, and 909 rclass identifications (Table 3 and Table S1). In addition, annotated functional descriptions and COG categories for genes/proteins were obtained.

**TABLE 3:** Overview of the KEGG annotation amounts curated in the IGTCM database.

Annotation by eggNOG‐mapper	Non‐redundant amounts	Protein amounts	Data resources
Enzymes	1677	630650	Integrated from KEGG database
GOs	16519	1509269	
KEGG_Module	357	349500	
KEGG_ko	5763	1442395	
KEGG_Pathway	830	898065	
BRITE	58	1442395	
KEGG_Reaction	2518	371700	
KEGG_rclass	909	353554	

Abbreviations: IGTCM, Integrative Genome Database of Traditional Chinese Medicine Plants; KEGG, Kyoto Encyclopedia of Genes and Genomes.

### 3.3 Browsing and searching the IGTCM database

The IGTCM database is a freely accessible web interface that can be accessed at http://yeyn.group:96/. Eight options are listed on the IGTCM top page: Home, Gene, Protein, RNA, Ingredient, Search, Tools, and About pages.

**FIGURE 3:**
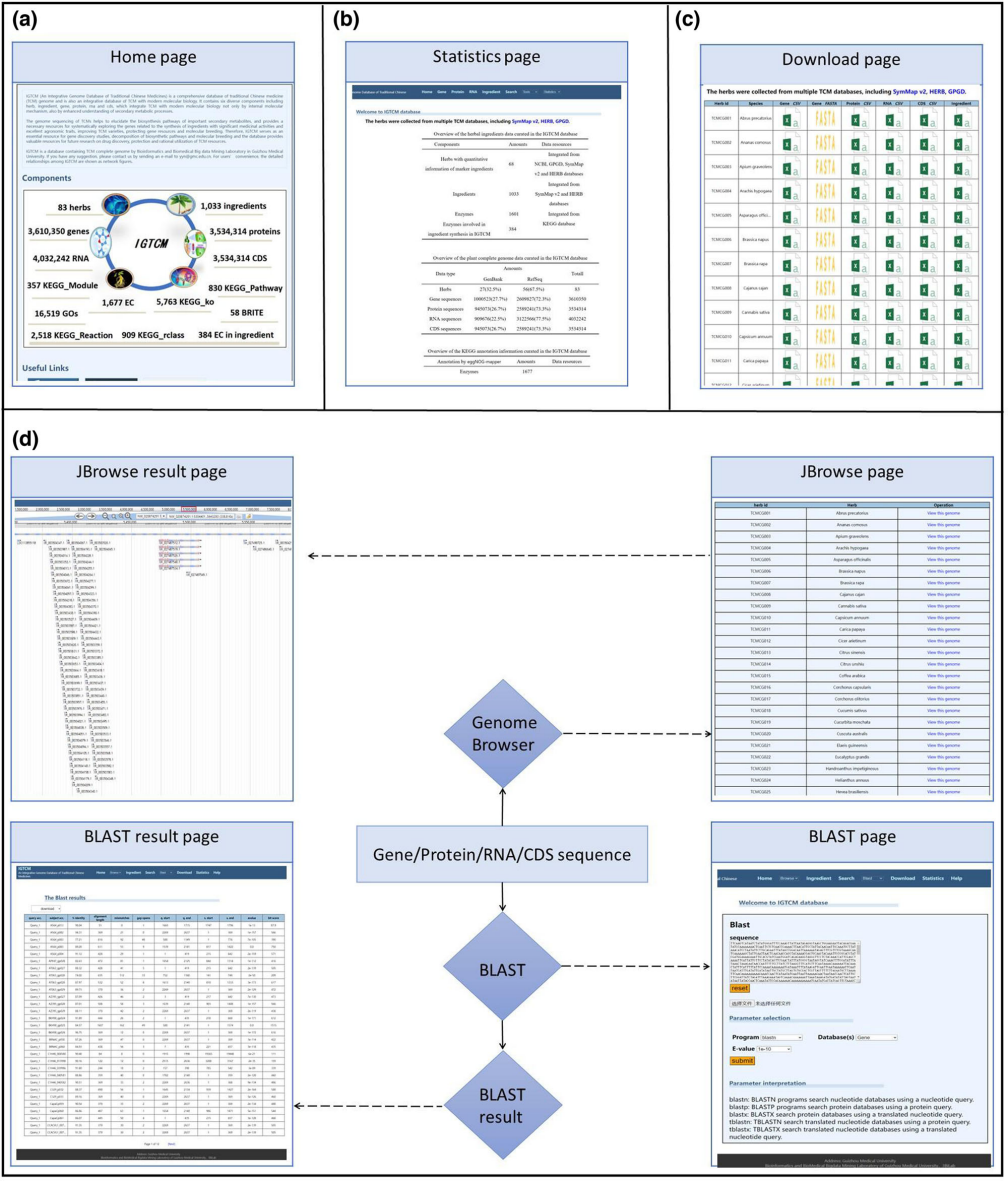
The IGTCM database interface. (a) The Home page contains an introduction, brief User's guide, data statistics, and location for feedback from users to the administrators. (b) The Statistics page provides multiple links for each herb, including links to genome visualizations, ingredients, genes, proteins, RNAs, and CDSs, as well as brief descriptions of the species and genera. (c) The Download page allows users to download the stored genome data. (d) The Tools page provides links to BLAST and JBrowse. An example of the usage process is shown. CDS, coding DNA sequence; IGTCM, Integrative Genome Database of Traditional Chinese Medicine Plants.

The Home page contains an introduction, brief User's guide, data statistics, and a location to leave feedback for the administrators (Figure 3a).

The About option has a dropdown menu that includes Statistics, Download, and Help pages. The Statistics page provides an overview and detailed information on the data stored in the IGTCM database. Multiple links are provided for each herb, including links to genome visualizations, ingredients, genes, proteins, RNAs, and CDSs; brief descriptions of the species and genera are also provided (Figure 3b). The Download page allows users to download data stored in the IGTCM database (Figure 3c). The Help page provides a detailed guideline for using the IGTCM.

The Tools page provides two tools: BLAST sequence similarity search and JBrowse genome visualization (Figure 3d).

**FIGURE 4:**
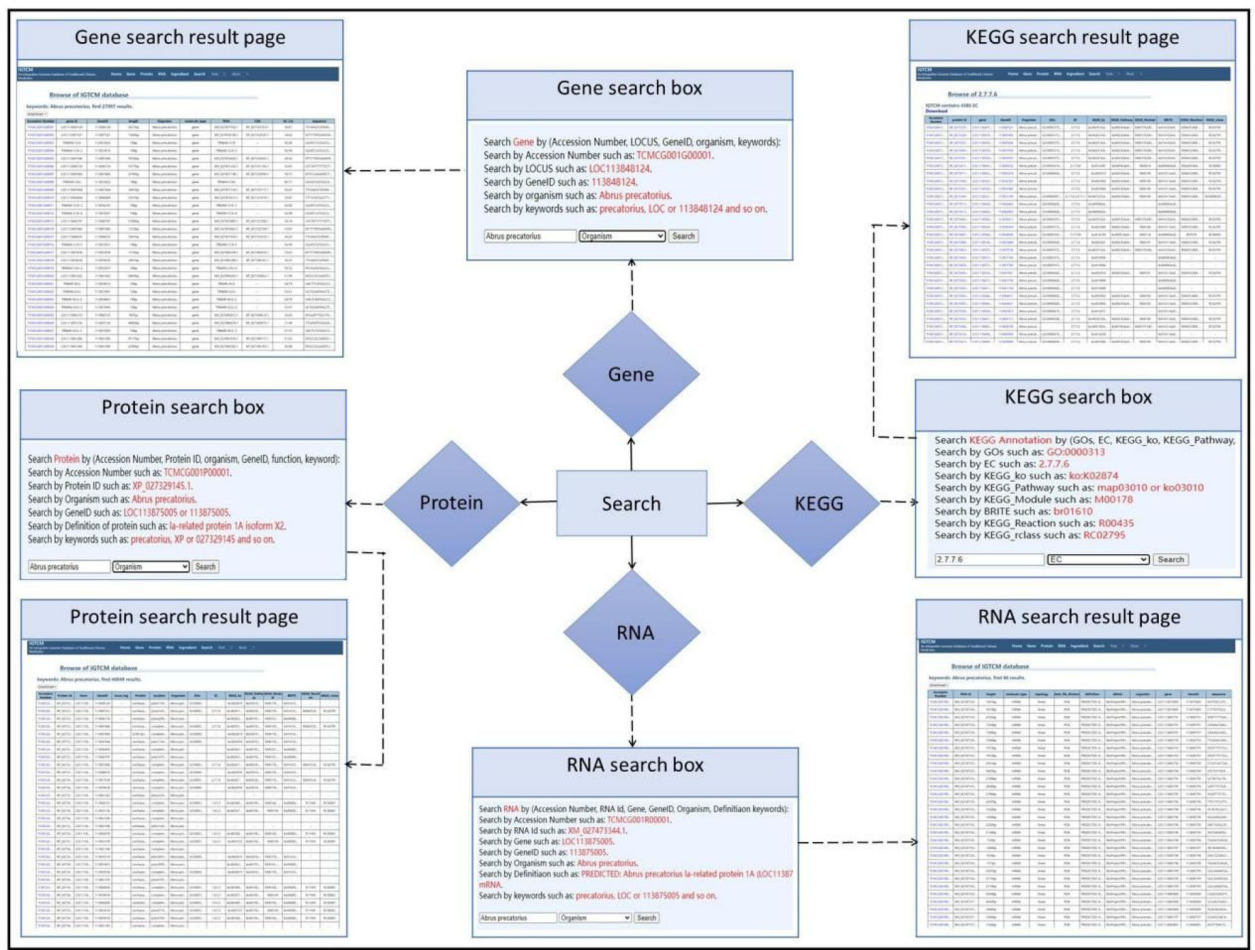
The IGTCM database interface. IGTCM offers four search boxes, gene, protein, RNA, and KEGG annotation information, each of which allows for an accurate search or a full database query based on the source of the data. Users can download search results or click links to relevant data pages. IGTCM, Integrative Genome Database of Traditional Chinese Medicine Plants; KEGG, Kyoto Encyclopedia of Genes and Genomes.

The Search page provides options to search the IGTCM database using herb name, gene, protein, RNA, CDS IDs, KEGG annotation, and keywords. Users can download the search results or link them to related data pages (Figure 4).

**FIGURE 5:**
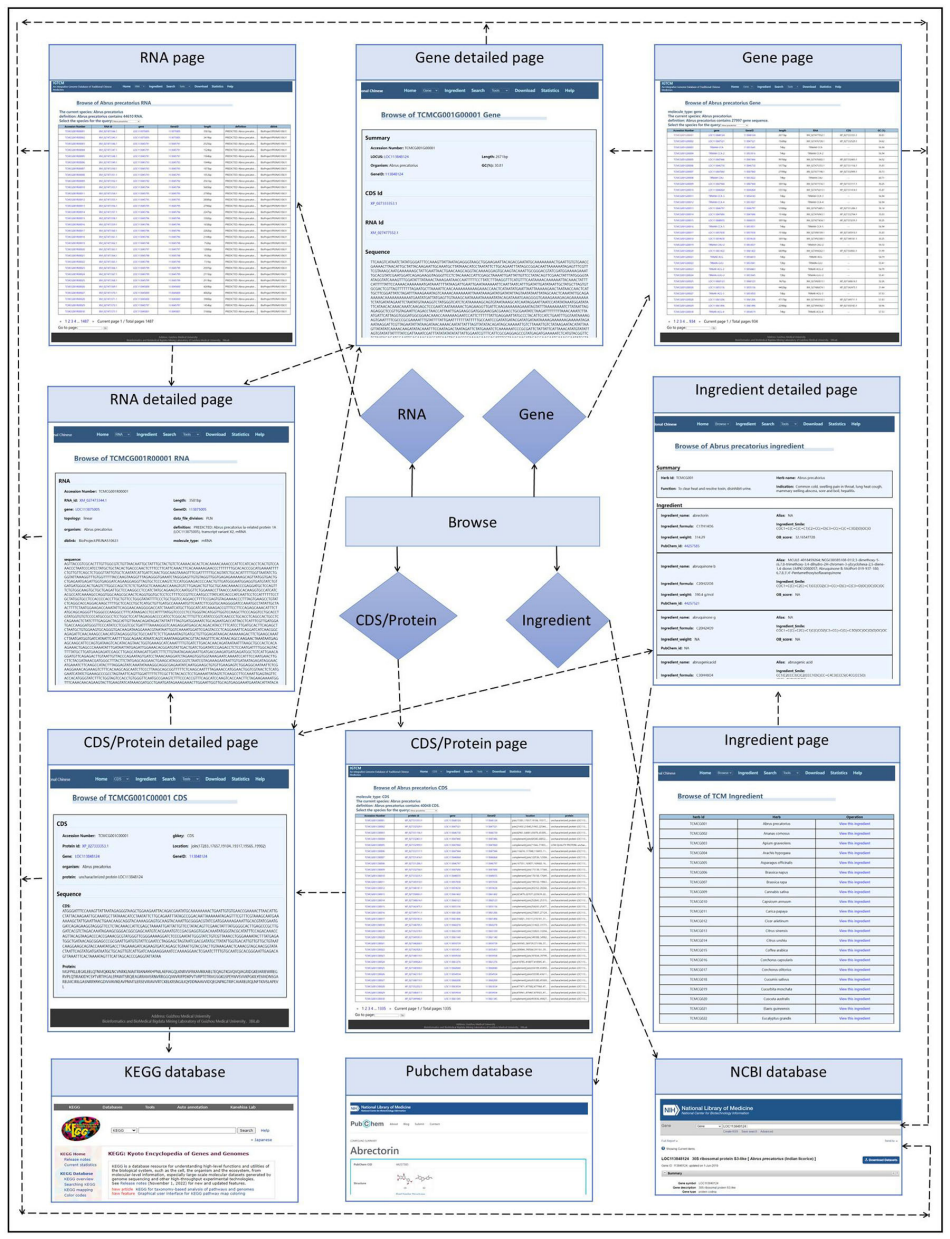
The workflow of the IGTCM database. Using an ID, gene, protein, and RNA data are linked to each other. The correlation between protein and RNA was realized using db_source. The correlations between genes/proteins and traditional Chinese medicine ingredients were determined through enzymes. KEGG annotation information was used to develop the cross‐species associations among traditional Chinese medicine. CDS, coding DNA sequence; IGTCM, integrative genome database of traditional Chinese medicine plants; KEGG, Kyoto Encyclopedia of Genes and Genomes; NCBI, The National Center for Biotechnology Information.

The browse option includes the Gene, Protein, RNA, and Ingredient pages, and it allows access to gene, protein, RNA, and compound information, respectively, in IGTCM. The data for these four tables are correlated, allowing the user to browse detailed molecular information. In addition, data between species have cross connectivity using KEGG annotated information. The Ingredient page provides a list of the ingredients for each herb in IGTCM, with links to detailed information about each ingredient and links across several species using enzyme classifications. The database integrates multiple types of information for each herb, and it provides links to external websites that can provide additional information (Figure 5).

### 3.4 Using the IGTCM database tools

The Tools page provides links to the JBrowse visualization of the genomic data of each herb in IGTCM, which allows gene information to be visually inspected in the genomic context. A link to BLAST is also provided on the Tools page, which allows users to align the genome sequences of herbs of interest. Users can also paste or upload FASTA sequences and use them as query sequences in BLAST searches against all the herb genomes in the IGTCM database (Figure 3d).

### 3.5 IGTCM usage case

**FIGURE 6:**
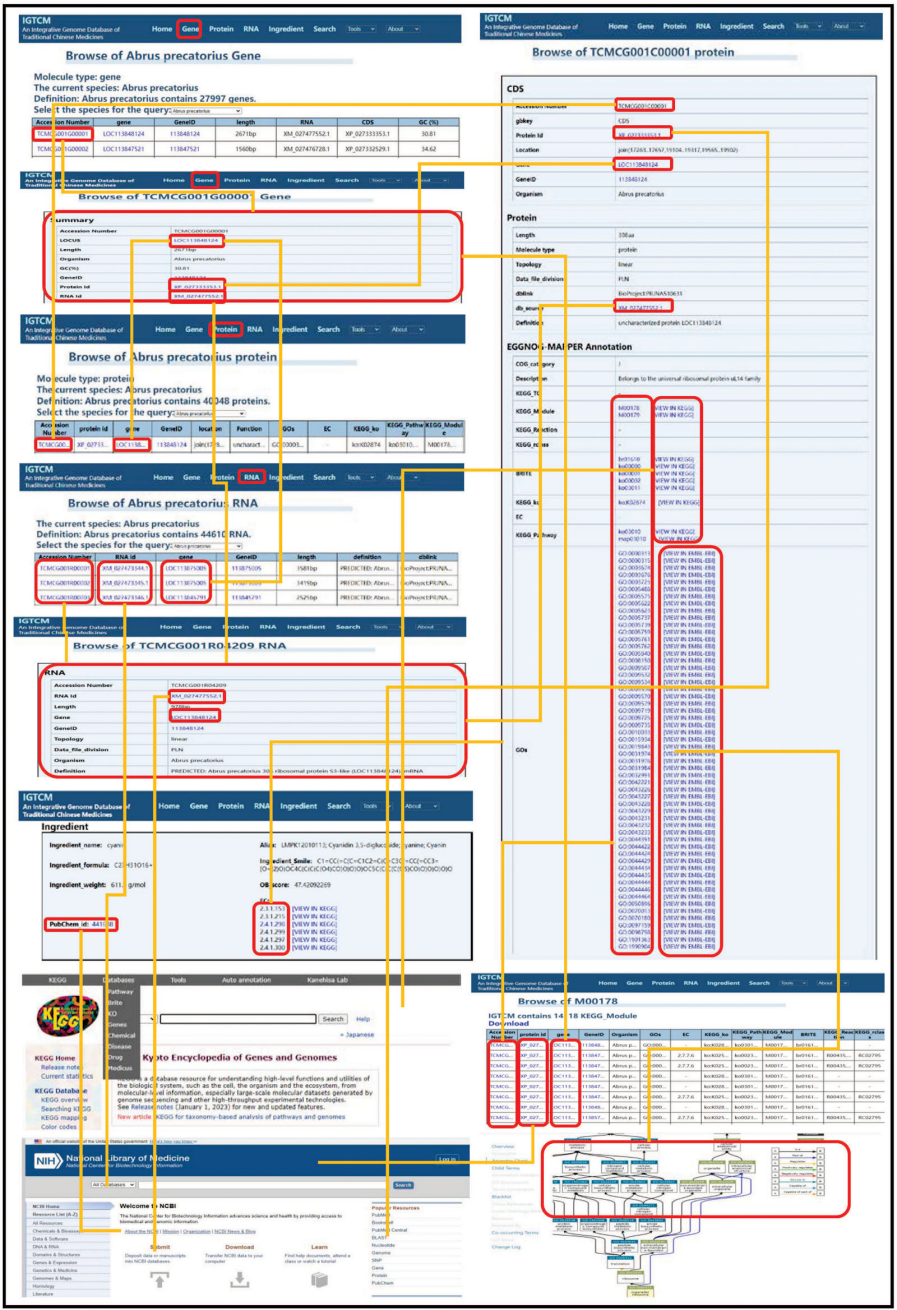
IGTCM usage case. IGTCM, Integrative Genome Database of Traditional Chinese Medicine Plants.

Indian licorice (*A. precatorius*) (Hovde et al., 2019) is a eudicot species in the family Fabaceae (pea family) that is used to break fevers, release toxins, and stimulate urination. The complete genome sequence of *A. precatorius* was sequenced in 2019. We download genome data from the NCBI ftp site and component data from the HERB database. *Abrus precatorius* has 27,997 genes, 40,048 proteins, 44,610 RNAs, and 13 compounds. For a minimal interconnectivity, we use *A. precatorius* as an example to show the capabilities of the IGTCM (Figure 6).

First, the gene, protein, and RNA data of *A. precatorius* can be associated with each other by clicking the ID number. Queries for genes in the IGTCM database generally reveal detailed information, such as EC, GOs, pathways, and ortholog information. For example, by clicking on *TCMCG001G00001*, its detailed display page is shown. There, you can query the protein and RNA encoded by gene *LOC113848124*. Second, IGTCM provides EC, KEGG, BRITE, and GO annotations for genes, and these features provide linkage across several species. Clicking on *XP_0273333531.1* in the *TCMCG001G00001* detailed page displays an interface or clicking *TCMCG001C00001* in the Protein interface provides GenBank and KEGG annotation information for *XP_0273333531.1*. In this interface, clicking on *LOC113848124* allows the user to return to the gene interface, clicking on db_source leads to the RNA interface, and clicking on KEGG annotation information allows the user to query all relevant KEGG annotation information in IGTCM. The users can also download the query results. In addition, the TCM components formed by the protein can be queried through the related enzymes. Third, through the molecular formulae of the chemical components, all the enzymes involved in the reaction can be found in the KEGG database. Thus, associated genes are already linked with (some) compounds in the ingredient list through the EC classification. The formation of the TCM component methylacetate in *A. precatorius* involves Enzyme 6.2.1.1. By clicking Enzyme 6.2.1.1, all the corresponding genes/proteins in *A. precatorius* can be queried, thereby revealing the associations between TCM components and genes/proteins. The queried results can also be downloaded.

## 4 DISCUSSION

### 4.1 Comparison of IGTCM with other TCM‐related genomic databases

Several TCM‐related genomic databases have been published, including the Global Pharmacopoeia Genome Database (GPGD) (Liao et al., 2022), HMOD: An Omics Database for Herbal Medicine Plants (Wang et al., 2018), and the Brazilian Pharmacopoeia Genomic Database (BPGD) (Zhou et al., 2021). Although these databases contain sequence data of plants used in TCM, most of the sequences lack full annotation information, which limits their usefulness. For instance, the GPGD and BPGD mainly focus on the identification of TCM plant species. However, they contain only 49 and 24 species, respectively, with complete genome annotations. The HMOD has only 23 published genomes of TCM plants, including *Panax notoginseng* and other important species. Furthermore, the TCM BLAST merely provides genomic data of 36 TCM plant species, ignoring protein, CDS, and RNA sequences. An integrative database for traditional Chinese medicine plant genomes only provides a large amount of TCM plant genome descriptive information, such as genome size, sequencing platform, and article sources, and it lacks a genome download function. All of the above databases have limitations when used for researching TCM plant genomes.

To better facilitate research on TCM plants and enrich existing full‐service TCM plant genome databases, we developed IGTCM. The IGTCM database provides detailed annotation information on the genes, proteins, RNAs, and CDSs of 83 herbal species used in TCM, and JBrowse can be used to visualize the genomes. GPGD, HMOD, and BPGD do not contain this type of detailed information. Information about the components of 68 of the 83 herbs is also provided in IGTCM. The IGTCM database will greatly support studies on the biological mechanisms and functions of the herbs used in TCM. Compared with the above databases, IGTCM has more genomic data on TCM plants. In addition, it includes protein, CDS, and RNA sequences of TCM plants for BLAST searches. Thus, it displays genes, proteins, RNAs, and CDSs of each TCM plant, allowing for integrative research.

### 4.2 Applications of the IGTCM database

The assembly and annotation of herbal genomes, as well as the systematic analyses of gene functions, will provide genetic information for building regulatory networks to elucidate the molecular mechanisms of herbal plants used as TCMs for disease prevention and treatment (Hu et al., 2019; Xin et al., 2019). In recent years, data on herbal genomes have rapidly accumulated, but these data are not well organized, mainly because of limitations such as non‐uniform formats and varied storage locations. In this work, we reanalyzed the annotated genomes of herbal species used in TCM that were available in NCBI's GenBank and RefSeq databases and integrated them with their components through database mining. All of these data were stored in IGTCM, a comprehensive database of herbal genomic and component data that has multiple applications in fields such as evolutionary history, model herb research, targeted herb breeding, and gene function/biosynthetic pathway analyses.

### 4.3 Novelty of the IGTCM database

The IGTCM database provides multiple data resources for herbal species used in TCM. It allows users to view genetically encoded products and TCM components. It also provides correlated KEGG annotation information, thereby facilitating the exploration of the potential mechanisms of action of genes with herbal components, as well as the identification of new candidate compounds for drug development.

The IGTCM database also provides high‐value gene, protein, RNA, and CDS information, based on manual screenings of plant genome data in the NCBI databases, for genome alignment, which fills the large gaps in TCM‐BLAST (Z. Chen et al., 2021) and GPGD, which do not contain comprehensive and abundant data. The BLAST tool in the IGTCM database can be used to search four genome databases (gene, protein, RNA, and CDS) using five different programs (blastn, blastp, blastx, tblastn, and tblastx). The sequence alignment results are displayed in a summary table. Users can view the herb genome sequences using JBrowse, which is embedded in IGTCM.

Furthermore, the IGTCM database provides a comprehensive list of herbs and their genomes and components, which offer a theoretical basis and an effective technological platform that can support the breeding, cultivation, and molecular identification of herbal species and facilitate the discovery of active components in TCM plants.

## AUTHOR CONTRIBUTIONS

**Yuan‐Nong Ye**: Funding acquisition; Supervision; Writing‐original draft. **Ding‐Fa Liang**: Data curation; Writing‐original draft. **Jia‐Hao Yi**: Validation. **Shuai Jin**: Data curation; Validation, **Zhu Zeng**: Conceptualization; Supervision; Writing‐review & editing.

## CONFLICT OF INTEREST STATEMENT

The authors declare that they have no competing interests.

## Supporting information

Supplement Table S1. Detailed statistics of the IGTCM database by species.
